# American Academy of Dental Science

**Published:** 1898-11

**Authors:** 

**Affiliations:** American Academy of Dental Science


### AMERICAN ACADEMY OF DENTAL SCIENCE.

The regular monthly meeting of the American Academy of Dental Science was held at Young's Hotel, Wednesday evening, February 2, 1898, at six o'clock.

A paper was read by Henry H. Burchard, M.D., D.D S.

(For Dr. Burchard's paper, see page 697.)

### DISCUSSION.

*Dr. Brackett.*—With such names on the programme as those of Drs. Jack, Kirk, Stellwagen, and Eames, I thought that any part which I might be called on to take in the discussion of this paper would be small, and would come after the others; but with the circumstances as they are, I consider it a privilege to begin the discussion, to state my appreciation of the paper itself, and to express my gratification that the conclusions, in most particulars, at which the writer arrives are quite in accord with what my own experience has been. I will quote two or three illustrative cases.

It is a fact that there come under the observation of the practitioner of dentistry, not very rarely, cases requiring great care and skill in diagnosis, and a capacity to practically apply such teachings as are impressed in this paper. There came to me about eleven o'clock in the forenoon of a day more than twenty years ago a gentleman of superior mental ability. He had been under the care of his medical adviser for two weeks for the treatment of neuralgia, located directly in the occiput. He had suffered extremely, and had been confined to his room for a considerable portion of the time, utterly incapacitated for giving attention to the practical affairs of life.

His medical adviser had suggested, everything else having failed to relieve him, that he came to me to have his teeth examined. On the right side of the lower jaw I found in the third molar an immense corono-buccal cavity, containing within a very broadly exposed, congested, and greatly irritated pulp. I said that I believed that was the occasion of his suffering. This seemed to him preposterous, but, when I explained how the nerves of the face and head could be affected by an irritated pulp, he said he would think about it. He went home and thought about it, and about two o'clock the next morning he came back. Gas was administered and the tooth was removed. When he had recovered from the effect of the anæsthetic his neuralgia was gone and there has been no return. That case is typical of numerous ones. Indeed, we usually find that patients coming into our offices with the statement that they have been having neuralgia have really been suffering from toothache.

Another case which I will mention was one in which there has been for years extreme general hypersensitiveness of the teeth Owing pretty certainly to a lack of good capacity for digestion, there is a rather prevalent acidity in the oral cavity, with a tendency of the teeth to decay all about the necks, an extreme sensitiveness to touch of an instrument, and an incapacity to eat acid fruits. Considerable suffering has been manifested in numerous instances after the insertion of a metallic filling of any sort.

Two or three years ago I filled with amalgam an extremely sensitive cervical cavity in a right lower second molar. There had been suffering previous to the operation, but no exposure of the pulp. After the operation, in addition to the hypersensitiveness of the dentine, there was what appeared to be earache, starting in the ear itself. It was so severe that at the end of twenty-four hours I said that the patient should come to Boston and try to secure the best advice that could be had with reference to the ear. She came and saw first Dr. Clapp, and was by him introduced to Dr. Blake, who made a most careful examination of the ear, and could make no other report than that he found everything normal. This satisfied me that the extreme sensitiveness of the dentine was the sum total of the cause of the suffering, which gradually lessened and disappeared entirely within a week or two.

There is a saying, worthy of being heeded, that "the man who is his own lawyer has a fool for a client." Sometimes a dentist, notwithstanding scores and hundreds of experiences to the contrary, thinks, if he is the patient, the patient knows which tooth is aching.

A few years ago I had, as I supposed, pain in a left upper molar. I hastily rushed to a brother dentist, and, without giving him the opportunity to make an examination, I asked him to make an application to the left upper molar, which relieved me for a time; but in a few days the pain came again, and I had another application made; and that continued for about six weeks, when my good friend suggested that he make an examination of the other teeth in my mouth. On doing so he found an exposed pulp in the left lower second molar, which had been the real seat of the trouble. I had not capacity enough to make out in that length of time where the pain was located.

It should not be forgotten, in our examination and in our dealing with cases as they come to us for the relief of pain, that the patient's statements, while they should be listened to and accepted as helpful, are not usually to be looked upon as of so much value as the results of our own investigation. In the matter of extraction, patients often come to our chairs with the statement that a particular tooth is the seat of pain. If we extract that tooth without demonstrating to our own satisfaction that it is the cause of the trouble, a very material percentage of cases of mistake will occur. It has sometimes seemed to me that twenty-five per cent. of error would be made through operating with the patient's testimony alone as the guide. These chances for error come through two or more channels. One of these is the well-recognized tendency of a nerve, under certain circumstances, to refer to the peripheral distribution the consequences of an irritation which is applied anywhere along the course of the nerve. The result of this is that an irritation which is taking place in a third molar seems to the patient to be located perhaps in the cuspid. We shall find numerous illustrations of this tendency if we look for them, so that I have made it a custom not to extract a tooth near the front of the mouth until I have satisfied myself about the conditions farther back. As a rule, unless a tooth shows unmistakable signs that it is the source of the patient's trouble, such as the presence of pericementitis, or alveolar abscess, or a glaringly exposed pulp, I look also on the opposite jaw, being influenced by such conditions as I have suggested, and because there are many cases in which the occlusion of even a single tooth is of very great consequence. Another reason why we should not depend entirely upon the words of the people who come to us to be relieved of pain is that their statements are not always ingenuous.

It was my privilege several years ago to have referred to me for dental treatment a good many patients of one of the most eminent practitioners of gynæcology of New England, now for some years out of practice. It was one of the cardinal principles of this specialist's practice to insist on the mouth being put in order, if the patient's condition would permit it, previous to his treatment of the pelvic organs or coincidently with that treatment. He recognized the possibility of harmful effects to the pelvic organs through the reflex action of any irritation which might affect the delicate nerve-filaments that are within the dentist's province.

If we were to allow, for an instant only, discussion of the converse of this paper,—of how disturbances in other regions may react most unfavorably upon the teeth,—I should have emphatic testimony to present. There comes to my mind another maxim of the specialist to whom I have just alluded,—that all surgical operations are most advantageously performed after the conclusion of the menstrual period. Not only operations within the gynæcologist's special surgery, but operations in general surgery as well, are most hopefully and helpfully accomplished during what Dr. Storer denominated the menstrual ebb. A case to which I wish to refer was that of a patient who came to me with trouble with the upper jaw. She was in a low systemic condition and very nervous. Examination showed a cavity in a left upper third molar. It was the only tooth in that region having a cavity, and the cavity was very small, almost literally a pinhead cavity. It was extremely sensitive, so much so that the drying of the cavity with bibulous paper caused greater shrinking than is usual if an instrument is put into the pulp-tissue. Although I had been conversant with the increased sensitiveness of the female nervous system at certain times, this impressed me as the most extreme instance I had ever known. She made it plain to me that she was then "unwell." I told her that if we could devise some palliative treatment for the time, after a while we could get on better with the excavation and filling of the cavity. There was put into the cavity deliquesced carbolic acid, and no further attempt was made to excavate until after menstruation had ceased. She was then feeling very much better systemically, and the preparation of the cavity was practically painless. I attribute the great change partly to the effect of the deliquesced carbolic acid, but more to the different systemic conditions of the patient.

In one of the opening sentences of the paper there is a reference to epilepsy, and the suggestion of the possibility of the disease being aggravated by the presence of an abnormal condition of the teeth. Very shortly after my graduation, I was consulted by a young man, perhaps twenty years of age, who was subject to epilepsy, and I was asked if I thought the teeth had anything to do with it. The right upper cuspid had never appeared. I was able, by diligent search, to locate it, deeply embedded at an angle of about forty-five degrees from the position it should have occupied, and with no room in the oral cavity for its eruption. In that case I had no opportunity to operate, but I believe that the impacted tooth was at least an aggravation of the trouble from which he suffered.

One of the points in the paper which specially interested me was in regard to the employment of different degrees of temperature in the diagnosis of obscure cases. Heat is of very great value as a test for diseased pulp tending to die in a confined space,—that is, in a chamber that is not perforated by open caries. In a case of sharp pain with intermissions the application of heat, causing marked increase of pain, may be the means of locating the seat of the trouble and indicating the treatment to be pursued.

A very satisfactory means of applying the heat-test is by using mineral talc, or French chalk, similar to that which the tailor uses for marking cloth. A piece of the material is easily cut into a convenient shape for carrying in a port-polisher so as to reach any tooth. It readily absorbs the heat of an alcohol lamp, may be heated hundreds of times, and does not corrode.

I agree cordially with the expression in the paper that calcifications within the pulp are not frequent causes of disturbances. I remember that our worthy president, some years ago, gave us a very interesting illustrative paper with reference to the very great frequency of these calcifications in the pulp-chamber. I believe that in some thousands of extracted teeth which he opened he found these calcifications present in about twenty per cent. The teeth were a miscellaneous collection, extracted for all reasons, and the showing appears to be that calcifications in pulp-tissue are much more frequent than is trouble from them.

In this connection a most important matter has not had mention,—that is, that in quite a percentage of true neuralgic affections the circumstance of nutrition is one of great consequence. I have seen a half-dozen cases, perhaps, of obscure, intractable neuralgia in elderly people in limited circumstances, poorly nourished, insufficiently clothed, not well housed, living in an unfavorable environment, perhaps overworked, in which I believed the affection was purely a systemic one. And we have heard of some cases under similar circumstances in which tooth after tooth has been extracted, until the patient has become edentulous, and the neuralgia has continued just the same. Of course there are opportunities for other nerve lesions outside of the province of the dentist. This fact should be recognized, and we ought not rashly to sacrifice teeth on the slight chance that relief may be afforded. In my own case overwork carried too far, and without proper sleep and taking of food, develops slight neuralgia of the scalp. I never have neuralgia under other circumstances. I have also the testimony of a physician who was at one time an extreme sufferer from neuralgia, sometimes affecting the side of the face, and again the supraorbital region; and being unable to locate the cause, or to find it through an examination of the teeth or eyes by competent specialists, after a very careful, searching investigation of his whole physical condition he came to attribute it to a lack of good functioning in the stomach, and he has found that by a very careful attention to his diet, eating regularly, and keeping himself in a well-nourished condition, his neuralgia is very much better.

This is a very prolific study, and one which we should all pursue for ourselves, in order that our patients may have the benefit of the most skilled treatment which it is in the power of the dentist to give.

*Dr. Eames.*—I hesitate a good deal about saying anything, especially as the essayist has covered the ground carefully, and Professor Brackett has given detailed cases, illustrating quite fully the principles upon which reflex disturbances depend. I recall some cases of reflex disturbance, but they offer no different principles from those which have been illustrated both in this paper and in cases already mentioned.

I have one case under care and study at the present time, that I might well mention and report upon in the future, if it ever becomes possible for me to ascertain the disturbing factor in the case. It is the case of a girl sixteen years old, whose teeth have been under my care from infancy, and in whose teeth there are no deep cavities, for the slightest appearance of decay has been checked. I can find no lesion whatever about the mouth. The girl is extremely robust and healthful in appearance, very large and strong, and she takes part in all the vigorous exercises and games of the girls of her age. It is my belief that the predisposing cause of the neuralgic pain from which she suffers is due mainly to this condition of plethora just described; besides, she is at that period of development and growth in which there is unusual activity and excitability. If there is hypertrophy of the cementum, or if there is, at that early age, a developing wisdom-tooth which is giving pressure, or if there is any calcific formation in the pulp, I cannot find any evidence which will enable me to ascertain if that be the fact. I do know that she has gouty ancestry, or that in many of her relatives pulp-nodules have been found.

In discussing this subject we are reminded that there are cases of reflex disturbance in which there is a distinct dental lesion which can be found and the cause removed; there are also cases in which the cause cannot be found by any ordinarily careful examination, and in some of the cases the Röntgen ray is of great service,—for instance, in such cases as hypertrophy of the cementum, impacted teeth, unerupted teeth, etc.,—and the paper does well in calling attention to this means of diagnosis. Cases in which there is no dental lesion to be found, and in which the pain is probably quite remote from the irritation, indicate some constitutional disturbance, and therefore the only hope of relief lies in constitutional treatment.

Many cases have been cited here to-night of the existence of pulp-nodules with no symptoms whatever, and I have found such. I have also found many cases, especially those in which there was a gouty diathesis, in which the pulp-nodule has seemed to give rise to extreme neuralgic pain. It has been stated in the paper that neuralgic pain may be due to pulp-nodules or other calcifications pressing upon nerve-fibres in the pulp. I do not see how any calcification within the pulp-tissue can occur without pressure upon the nerve-fibres. Therefore, as we have reason to believe that there are many cases in which no response is made to the pressure of the pulp nodule, I conclude that in the cases in which it does give intense pain it is due to the general condition. The nervous system is in such an irritable condition that the mere presence of the nodule produces intense pain. In such cases our only hope for relief is in constitutional treatment; the patient must be put into a condition similar to that of those who have pulp-nodules without pain.

*Dr. Jewell.*—I would like to say just a word in regard to a case I had. The patient, a lady about sixty years of age, was sent to me some time ago by a physician. She had been having a great deal of neuralgic pain in her lower jaw. After a thorough examination I told her I could not find anything that would cause the trouble; that her teeth were all perfectly sound. She said she thought the cause might be the second molar, which had a small gold filling, but it appeared to be all right. In a few days she came back to me and insisted that the pain was in that second molar tooth, so I removed the gold filling and replaced it with gutta-percha.

The pulp was not exposed; in fact, the cavity was not very near the pulp. That change afforded her a little relief, but not complete relief, and I then removed the gutta-percha and cut down near the pulp, and found it extremely sensitive. I then made an application to destroy the pulp, which was not very successful. When she came in again I opened into the pulp-chamber and found a very large pulp-stone almost filling the entire chamber. Of course, the pulp was extremely sensitive, and I tried to allay the irritation by opening into it freely, so as to make certain of destroying it, and even then did not succeed in removing the sensation. Finally, as a last resort, I removed the tooth, and she got some little relief, but still the neuralgic trouble continued in a very severe form; in fact, she has it constantly. The query in my mind now is, how far I should be justified in opening into or extracting the remaining teeth.

She is an intense sufferer from neuralgia, and there is no doubt in my mind but that it proceeds from her apparently sound teeth. I think they are all or a part of them in the same condition.

*Dr. Eames.*—I would like to ask Dr. Jewell if in this case he was able to drill down into the roots to any extent?

*Dr. Jewell.*—Not fully. They were nearly closed up.

*Dr. Eames.*—I would simply suggest that sometimes these deposits extend quite a distance into the root-canal. In some cases, when seemingly the path is entirely calcified, if we penetrate farther into the canal we shall come in contact with living pulp-tissue. It may be it is the irritation of this nerve-tissue which is left which is the cause of the trouble.

*Dr. Jewell.*—In this case the roots were almost completely closed up. What I would like to know is how to treat such a case. Would I be justified in drilling into the other sound teeth, or in extracting them? The patient is still a great sufferer, and I have no doubt of the cause. The pain is confined to the right side of the lower jaw. The upper teeth are in good condition, very hard and dense in character.

*Dr. G. T. Baker.*—The case is almost identical with one I had in my practice, only the relief was permanent after the pulp-chamber was opened and filled.

*Dr. Eames.*—While in many cases of severe neuralgia the cause may be traced to calcified formations in the pulp, which being removed effect a cure, yet in one case I found such a formation which had never given pain. In the case to which I refer I was obliged to remove a tooth for the purpose of regulating, and after doing so I broke it open and found a large pulp-nodule. I have no theory to offer regarding its presence there, except to mention that there were some appearances of gouty diathesis in the teeth. There had been no caries in the tooth, and no disease of the pericementum.

*Dr. Smith.*—A most marked case of reflex neuralgia resulting from calcification of the pulp occurred in my practice a few years ago. The patient was a man who was suffering with excruciating pain from the shoulder down through the forearm. His physician had kept him under the influence of morphine for a week before the case came to me, and they felt that the trouble was caused by an erupting wisdom-tooth. The gum had been lanced thoroughly and well, without any relief to the poor sufferer. Finally he was sent to my office one Sunday morning. The wisdom-tooth was not erupted, but there seemed to be plenty of room for it to erupt. The second molar was unfilled. The first molar had a small mesial cavity filled with cement. I diagnosed this case by eliminating from the diagnosis all other possible causes. The upper teeth were examined thoroughly, tested in every way, and I came to the conclusion that it might be a pulp-stone in this first molar. I explained to him what I thought, and that I proposed taking out the cement filling and making an investigation, giving him to understand that it might not be the cause of the trouble. I took out this cement filling, having to work very carefully on account of the extreme sensitiveness. The cavity did not extend near the pulp, but I continued to drill carefully until I made an exposure and found that a cornua of the pulp had calcified and detached itself, so that it acted like a shot put into the pulp-chamber. I removed the pulp-stone and dressed the cavity, and in two hours after he left my office he was free from all trouble in his shoulders and arm. I have had similar cases, but none where the relief was so prompt.

I am inclined to differ from those who think calcification of the pulp is not a frequent cause of neuralgia. Dr. Brackett has referred to the specimens examined by Dr. Cooke, stating that in the examination of a great number of extracted teeth, taken as they came, a large percentage of calcification of the pulp was found. Yet it seemed to me that while this investigation proved to us that the presence of calcific matter in the pulp was more common than we thought, it does not prove that patients who had those teeth had not suffered from neuralgia. I believe Dr. Cooke does not know the history of the teeth which he examined; they may have been the cause of treatment by physicians for some obscure trouble, and this trouble the physician may or may not have been successful in relieving. I feel, as I said before, that calcification of the pulp plays no small part in cases of neuralgia, the cause of which cannot be found, especially so where we have a cornua of the pulp calcified and detached, producing the effect of a foreign body in the pulp.

*Dr. Werner.*—We are not physicians enough to treat all cases of neuralgic condition. The perversions of the general functions of the body are, to my mind, many times the cause of neuralgia, and how we specialists can think our treatment intelligent or efficient by simply removing teeth I do not see. When an individual suffers from a thorough perversion of his systemic functions, with a decided uric acid diathesis, with an impairment of the whole eliminative system, composed of five or six miles of ducts, is it any wonder that a neuralgic condition exists, and how can such a condition be treated locally? Why not resort first to the treatment of the perverted constitutional condition? I can have neuralgia if I work too continuously at my chair, if I eat meat three times a day and take no exercise, and can localize it more or less in a first molar. When I exercise, when I get plenty of out-door work, I do not have neuralgia; and when I do not find myself able to take the necessary out-door exercise I resort to the lazy man's way of correcting systemic conditions,—take a Turkish bath, and with the improvement of the general tone my neuralgia vanishes. It would be folly for me to think that I ought to have my first molar out, or that I should suspect a troublesome calcification taking place in the pulp. No doubt calcification is going on, but unless it becomes very marked or I become much debilitated, I will not notice it. I have long since ceased to think that by simple dental interference you can correct many of those obscure cases of neuralgia.

*Dr. Andrews.*—I wish to add a word to one phase of this discussion. In the course of his remarks, Professor Smith referred to the large percentage of pulp-stone found in apparently healthy mouths. I do not see how there can be an abnormal calcification of the pulp in the mouth of a very healthy person. There must be some pathological condition present to produce this result. A cause of much obscure trouble is often overlooked in our endeavors to diagnose the case. I refer to malocclusion, and the clinching of teeth during sleep, and the consequent irritation. In such cases there is apt to be over-stimulation of some active cell-tissues, producing a growth of bone, exostosis or hyperostosis of the root, or an irritation from the side of the pulp-cavity, in which case calcific excretions or growths form and grow into the pulp-tissue. I cannot see how this can be considered other than a pathological condition, brought about by irritation of active cell-tissue whose deposits are increased by the constant irritation. I have had some curious cases in my own practice which I have succeeded in tracing to the grinding or clinching of teeth in sleep. There will be one or two teeth which will impinge upon some tooth in the opposite arch, causing an irritation of periosteal tissue. This is shown pretty conclusively by patients saying that when they wake up in the morning the tooth is sore and has a sort of dull ache, which disappears later. During this time the patient may also have neuralgic pains at some remote point. Generally, I have found that grinding the occluding tooth so that it cannot be touched by articulation has proved very beneficial. In one case I tried for quite a long time to treat a neuralgic trouble about the head and face. I asked the patient, after some considerable treatment, if he was in the habit of grinding his teeth. He said that he was not; but on another visit told me he found that when he was nervous he was in the habit of grinding on an upper cuspid, clinching it rather hard, and he had forgotten to tell me about it. On grinding the lower teeth a trifle, so that he could not articulate with the upper one, the recovery was almost immediate. I think the number of cases of obscure pain which might be traced to the grinding of the teeth during sleep is a great deal larger than we have any idea of.

*Dr. Brackett.*—It would be unjustifiable presumption in me to say concerning histology anything not in accord with our friend at that end of the table, yet it is my understanding that the calcification of a pulp is not an entirely unnatural process; that there is a steady deposit going on,—not markedly, as in some of the instances which Dr. Cooke reported in his investigations, but that calcification does go on until in elderly people, in some cases, the pulp is entirely obliterated. I have had several cases myself where a great amount of suffering was undeniably caused by calcifications in the pulp. One case was that of a lady who was in bed for two weeks, and was treated by a prominent New York physician for a severe neuralgia, and referred to me for examination of the teeth. I diagnosed pulp-stone in a molar, and was obliged to make several applications for the destruction of the pulp. After I got out quite a group of calcified nodules, relief came. I have had a few instances of obscure pain in sound teeth which, I have found out in a slow, blundering way, were occasioned by the dying of the pulp, without any explanation that I could find other than in one instance there was present a rachitic condition. The patient was a dwarf, and presented many physical defects. In that case I treated successfully four dying pulps in perfectly sound molar teeth. The history is much like that of a tooth that has been filled with metal close to the pulp. In my first case of this kind I was a long time in finding out that the trouble was caused by a morbific pulp. Another case was that of a lady in whose mouth I found a dying or dead pulp in each of four or five molars, and in every instance the teeth were sound or had been so previous to the dying of the pulp.

With reference to the matter of which Dr. Eames was speaking, a great many years ago there was brought to Dr. Riggs, of Hartford, a patient telling some such story as the young lady mentioned by Dr. Eames. This young lady was perhaps nineteen years of age. Her teeth were hard and strong, of excellent quality, and no cavities could be discovered on examination; but the patient was suffering a severe neuralgia, extending from shoulder to shoulder. Other dentists had made careful examinations without being able to discover any cause for the trouble, and the case was pronounced to be one of intractable neuralgia. Dr. Riggs said, on inspection of the case, that the trouble was undoubtedly caused by hypertrophy of the cementum. It seemed as if he knew by intuition. Anyway, his opinion was regarded of such value that his advice to remove from a fairly regular arch four perfectly sound bicuspids was followed. His diagnosis proved correct, for it was found that on the roots of the bicuspids exostosis of the cementum had taken place, and his theory that the relief of the crowding would be followed by a cessation of the suffering also proved to be the fact.

*Dr. Andrews.*—I was not referring to normal calcification of a pulp-cavity from the odontoblastic layer, which takes place evenly all over the cavity as age goes on, until in some cases two-thirds or nearly the whole cavity has become calcified. This is entirely different from the calcification which produces excretions which might be compared to a papilla of bone growing into the pulp-substance. I have several cases in my own collection of specimens where there are growths like papilla coming from the side of the pulp-cavity, and in some of the cases this abnormal calcification has grown to the extent of almost closing up the pulp-cavity. This calcific growth goes on independent of the odontoblasts, and is at the expense of the connective-tissue cells of the pulp-tissue, and is the result of some pathological condition, and not the normal growth from the whole odontoblastic layer, which, as I have said, may go on to the extent of almost closing up the pulp-cavity and yet be a perfectly normal and healthy condition.

*President Cooke.*—In regard to the percentages of teeth which I found containing pulp-stones: as I remember it, I cracked up about five thousand teeth, and out of that number I found over a thousand containing pulp-stones, varying in size. The largest percentage was found in the molars, which, I think, contained something like thirty per cent. The cuspids came next, with a little more than ten per cent., and the bicuspids and incisors were about the same, showing but a small percentage in each. I do not think that every time you have a pulp-stone you have neuralgia. It is my belief that, if I cracked up every extracted tooth I could find, I would get about the same percentages of pulp-stones that I found in those which I did examine. It has been suggested that we do not know the history of them. That is very true, and many of them may have caused trouble which the physician or dentist could not account for. I think it would be interesting if we could get statistics showing the number of cases in which we have treated neuralgia by the removal of the deposit called pulp-stone. I think they would be comparatively few, and yet I think the number of pulp-stones in the mouths of our patients is very great. In speaking with a specialist on nervous diseases with regard to this subject, he said it would be possible for the pulp-stones to be present, and while the tone of the body was up a person would feel no inconvenience from them, but if the nervous system should in any way become run down, then you would be likely to get twinges from the teeth. They might be the point most easily irritated. That is his theory.

When I was making investigation, I became so familiar with the peculiarities of teeth that in looking them over I would say to myself, "There is a pulp-stone in that tooth," and I would find one in almost every instance. In some cases they would be small, like shot, and in other cases would almost entirely fill the pulp-cavity. I remember one lower third molar which I cracked open and found the pulp entirely calcified, but was movable in the cavity. In cases of decayed teeth, it has seemed to me that the calcific matter has been thrown in not so much to protect the pulp from decay, but has probably been caused by the irritation of the decay. I sent about three hundred specimens of the different kinds down to the Harvard Dental School, where any one who is interested may examine them.

Most of the teeth that we are called upon to treat are imperfect in some way, and, taking into consideration the conclusion which I reached in my investigation,—that the percentage of pulp stones is about the same in all teeth,—in the treatment of any obscure trouble in which the teeth are suspected, I should recommend trying everything possible in the way of local and systemic treatment, and finally treating for pulp-stone.

William H. Potter, D.M.D.,
*Editor American Academy of Dental Science.*

